# PMab-235: A monoclonal antibody for immunohistochemical analysis against goat podoplanin

**DOI:** 10.1016/j.heliyon.2019.e02063

**Published:** 2019-07-10

**Authors:** Yoshikazu Furusawa, Shinji Yamada, Takuro Nakamura, Masato Sano, Yusuke Sayama, Shunsuke Itai, Junko Takei, Hiroyuki Harada, Masato Fukui, Mika K. Kaneko, Yukinari Kato

**Affiliations:** a Department of Antibody Drug Development, Tohoku University Graduate School of Medicine, 2-1 Seiryo-machi, Aoba-ku, Sendai, Miyagi, 980-8575, Japan; b New Industry Creation Hatchery Center, Tohoku University, 2-1 Seiryo-machi, Aoba-ku, Sendai, Miyagi, 980-8575, Japan; c ZENOAQ RESOURCE CO., LTD., 1-1 Tairanoue, Sasagawa, Asaka-machi, Koriyama, Fukushima, 963-0196, Japan; d Department of Oral and Maxillofacial Surgery, Graduate School of Medical and Dental Sciences, Tokyo Medical and Dental University, 1-5-45, Yushima, Bunkyo-ku, Tokyo, 113-8510, Japan

**Keywords:** Lymphatic endothelial cells, PMab-235, Goat podoplanin, PDPN

## Abstract

Sensitive and specific monoclonal antibodies (mAbs) against not only human but also mouse, rat, rabbit, dog, cat, bovine, pig, and horse podoplanins (PDPNs) have been established in our previous studies. However, anti-goat PDPN (gPDPN) has not been established yet. PDPN has been utilized as a lymphatic endothelial cell marker especially in pathological diagnoses; therefore, mAbs for immunohistochemical analyses using formalin-fixed paraffin-embedded tissues are needed. Although we recently demonstrated that an anti-bovine PDPN mAb, PMab-44 cross-reacted with gPDPN, PMab-44 did not detect lymphatic endothelial cells in immunohistochemistry. In this study, we immunized mice with gPDPN-overexpressing Chinese hamster ovary (CHO)–K1 (CHO/gPDPN) cells, and screened mAbs against gPDPN using flow cytometry. One of the mAbs, PMab-235 (IgG_1_, kappa), specifically detected CHO/gPDPN cells by flow cytometry. Furthermore, PMab-235 strongly detected lung type I alveolar cells, renal podocytes, and lymphatic endothelial cells of colon by immunohistochemistry. These findings suggest that PMab-235 may be useful as a lymphatic endothelial cell marker for goat tissues.

## 1 Introduction

Podoplanin (PDPN)/T1alpha/Aggrus is a type I transmembrane sialo-glycoprotein, which is expressed in many cell types, such as pulmonary type I alveolar cells, renal podocytes, mesothelial cells, and epithelial cells or lymphatic endothelial cells of many organs [1]. PDPN could induce platelet aggregation by binding to the endogenous receptor of PDPN, C-type lectin-like receptor-2 (CLEC-2) [2]. PDPN has been used to distinguish lymphatic endothelial cells from vascular endothelial cells in pathophysiological studies [3]. The PDPN-CLEC-2 interaction facilitates the separation of lymphatic vessels and embryonic blood [4]. Our previous analyses of glycopeptides produced by Edman degradation and mass spectrometry demonstrated that the disialyl-core1 (NeuAcα2-3Gal β1-3(NeuAcα2-6)GalNAcα1-O-Thr) structure was attached to a glycosylation site at residue Thr52 of human PDPN (hPDPN) [5]. Sialic acid-deficient PDPN recovered its activity after additional sialylation, indicating that the sialylated core1 of Thr52 is critical for PDPN-induced platelet aggregation.

The expression of hPDPN has been reported in many malignant tumors, including malignant oral squamous cell carcinomas [6], lung squamous cell carcinomas [7], esophageal squamous cell carcinomas [8], malignant mesotheliomas [9], osteosarcomas [10], chondrosarcomas [11], brain tumors [12], and seminomas of testicular tumors [13]. Our previous studies demonstrated that PDPN expression in Chinese hamster ovary (CHO)–K1 cells promoted pulmonary metastasis in both an experimental and a spontaneous mouse model [14]. No differences in the size of metastatic foci or in primary tumor growth were found in either set of mice. PDPN-expressing cells, which were covered with platelets, were found to be arrested in the lung microvasculature 30 minutes after injection. Furthermore, lung metastasis resulting from PDPN expression decreased the survival of the mice. We showed that point mutation at the platelet aggregation-stimulating (PLAG) domain of hPDPN lost both platelet aggregation and metastasis. Inhibition of platelets with aspirin reduced the formation of PDPN-promoted metastasis, indicating that PDPN contributes to the establishment of metastasis by promoting platelet aggregation without affecting subsequent growth.

We have developed monoclonal antibodies (mAbs) against not only human [12], mouse [15], rat [16], rabbit [17], but also bovine [18], dog [19], cat [20], pig [21], horse [22], Tasmanian devil [23], alpaca [24], tiger [25], and bear [26] PDPNs. About anti-hPDPN mAbs, we established cancer-specific mAbs (CasMabs), such as LpMab-2 [27] and LpMab-23 [28,29]. An anti-dog PDPN (dPDPN), PMab-38 also showed cancer-specificity [19, 30, 31]. PMab-44 (an anti-bovine PDPN (bovPDPN) mAb), PMab-52 (an anti-cat PDPN (cPDPN) mAb), PMab-213 (an anti-pig PDPN (pPDPN) mAb), PMab-219 (an anti-horse PDPN (horPDPN) mAb), PMab-233 (an anti-Tasmanian devil PDPN (tasPDPN) mAb), PMab-225 (an anti-alpaca PDPN (aPDPN) mAb), PMab-231 (an anti-tiger PDPN (tigPDPN) mAb), and PMab-247 (an anti-bear PDPN (bPDPN) mAb) have been shown to be useful for flow cytometry, Western blot, and immunohistochemical analyses [18, 20, 21, 22, 23, 24, 25, 26]. In this study, we immunized mice with CHO/goat PDPN (gPDPN) cells and established mAbs against gPDPN.

## 2 Materials and methods

### 2.1 Cell lines

Ch 1 Es, a fibroblastic cell line from normal fetal goat esophagus was obtained from the Japanese Collection of Research Bioresources Cell Bank (Osaka, Japan). P3X63Ag8U.1 (P3U1) and CHO–K1 cells were obtained from the American Type Culture Collection (ATCC; Manassas, VA, USA). Synthesized DNA (Eurofins Genomics KK, Tokyo, Japan) encoding gPDPN (accession No.: XM_005690821.3) plus an N-terminal MAP16 tag (PGTGDGMVPPGIEDKI), which is recognized by an anti-MAP16 tag mAb (PMab-1: the same mAb against MAP tag [32]), was subcloned into a pCAG-Neo vector (FUJIFILM Wako Pure Chemical Corporation, Osaka, Japan). Plasmids were transfected using Lipofectamine LTX with Plus Reagent (Thermo Fisher Scientific Inc., Waltham, MA, USA). Stable transfectants were selected by limiting dilution and cultivation in a medium containing 0.5 mg/ml of G418 (Nacalai Tesque, Inc., Kyoto, Japan).

The P3U1, CHO–K1, CHO/gPDPN, CHO/hPDPN [33], CHO/mouse PDPN (mPDPN) [33], CHO/rat PDPN (rPDPN) [16], CHO/rabbit PDPN (rabPDPN) [17], CHO/dPDPN [19], CHO/bovPDPN [18], CHO/cPDPN [20], CHO/pPDPN [21], CHO/horPDPN [22], CHO/tigPDPN [25], CHO/aPDPN [24], CHO/bPDPN [26], CHO/tasPDPN [23], CHO/sheep PDPN (sPDPN) [34], and CHO/whale PDPN (wPDPN) [35] were cultured in a Roswell Park Memorial Institute (RPMI) 1640 medium (Nacalai Tesque, Inc.), supplemented with 10% of heat-inactivated fetal bovine serum (FBS; Thermo Fisher Scientific Inc.), 100 units/mL of penicillin, 100 μg/mL of streptomycin, and 25 μg/mL of amphotericin B (Nacalai Tesque, Inc.). Ch 1 Es was cultured in E-MEM with Earle's Salts, L-Gln and Non-Essential Amino Acids (Nacalai Tesque, Inc.), supplemented with 10% of heat-inactivated FBS (Thermo Fisher Scientific Inc.), 100 units/mL of penicillin, and 100 μg/mL of streptomycin (Nacalai Tesque, Inc.). The cells were grown in an incubator at 37 °C with humidity and 5% CO_2_ and 95% air atmosphere.

### 2.2 Hybridoma production

Female BALB/c mice (6 weeks of age) were purchased from CLEA Japan (Tokyo, Japan). The animals were housed under specific pathogen-free conditions. The Animal Care and Use Committee of Tohoku University approved all animal experiments. We used a Cell-Based Immunization and Screening (CBIS) method [21, 22, 23, 24, 25] to develop mAbs against gPDPN. Briefly, one BALB/c mouse was immunized with CHO/gPDPN cells (1 × 10^8^) intraperitoneally (i.p.) together with the Imject Alum (Thermo Fisher Scientific Inc.). The procedure included three additional immunizations, followed by a final booster injection administered i.p. 2 days prior to the harvest of spleen cells. Subsequently, these spleen cells were fused with P3U1 cells using PEG1500 (Roche Diagnostics, Indianapolis, IN, USA), and the hybridomas were grown in an RPMI medium supplemented with hypoxanthine, aminopterin, and thymidine (HAT) for selection (Thermo Fisher Scientific Inc.). The culture supernatants were screened by flow cytometry.

### 2.3 Flow cytometry

The cells were harvested following a brief exposure to 0.25% trypsin and 1 mM ethylenediaminetetraacetic acid (EDTA; Nacalai Tesque, Inc.). The cells were washed with 0.1% bovine serum albumin (BSA) in phosphate-buffered saline (PBS) and treated with primary mAbs for 30 min at 4 °C. Thereafter, the cells were treated with Alexa Fluor 488-conjugated anti-mouse IgG (1:2000; Cell Signaling Technology, Inc., Danvers, MA, USA) or Oregon Green anti-rat IgG (1:2000; Thermo Fisher Scientific Inc.). Then, fluorescence data were collected using the SA3800 Cell Analyzer (Sony Corp., Tokyo, Japan).

### 2.4 Determination of binding affinity by flow cytometry

CHO/gPDPN was suspended in 100 μL of serially diluted PMab-235. Then, Alexa Fluor 488-conjugated anti-mouse IgG (1:200; Cell Signaling Technology, Inc.) was added. Fluorescence data were collected using the EC800 Cell Analyzer (Sony Corp.). The dissociation constant (*K*_D_) was calculated by fitting the binding isotherms to built-in one-site binding models in the GraphPad PRISM 6 (GraphPad Software, Inc., La Jolla, CA, USA).

### 2.5 Immunohistochemical analyses

Goat tissues were collected in NIPPON ZENYAKU KOGYO CO., LTD. (Fukushima, Japan), fixed in 4%-Paraformaldehyde Phosphate Buffer Solution (Nacalai Tesque, Inc.). Histological sections (4-μm thick) were directly autoclaved in a citrate buffer (pH 6.0; Nichirei Biosciences, Inc., Tokyo, Japan) for 20 min, and were blocked using the SuperBlock T20 (PBS) Blocking Buffer (Thermo Fisher Scientific Inc.), incubated with PMab-235 (1 μg/mL) or PMab-44 (1 μg/mL) for 1 h at the room temperature, and then treated with the Envision + Kit for mouse (Agilent Technologies Inc. Santa Clara, CA, USA) for 30 min. Color was developed using 3,3′-diaminobenzidine tetrahydrochloride (DAB; Agilent Technologies Inc.) for 2 min, and counterstaining was performed using hematoxylin (FUJIFILM Wako Pure Chemical Corporation).

## 3 Results

**Fig. 1:**
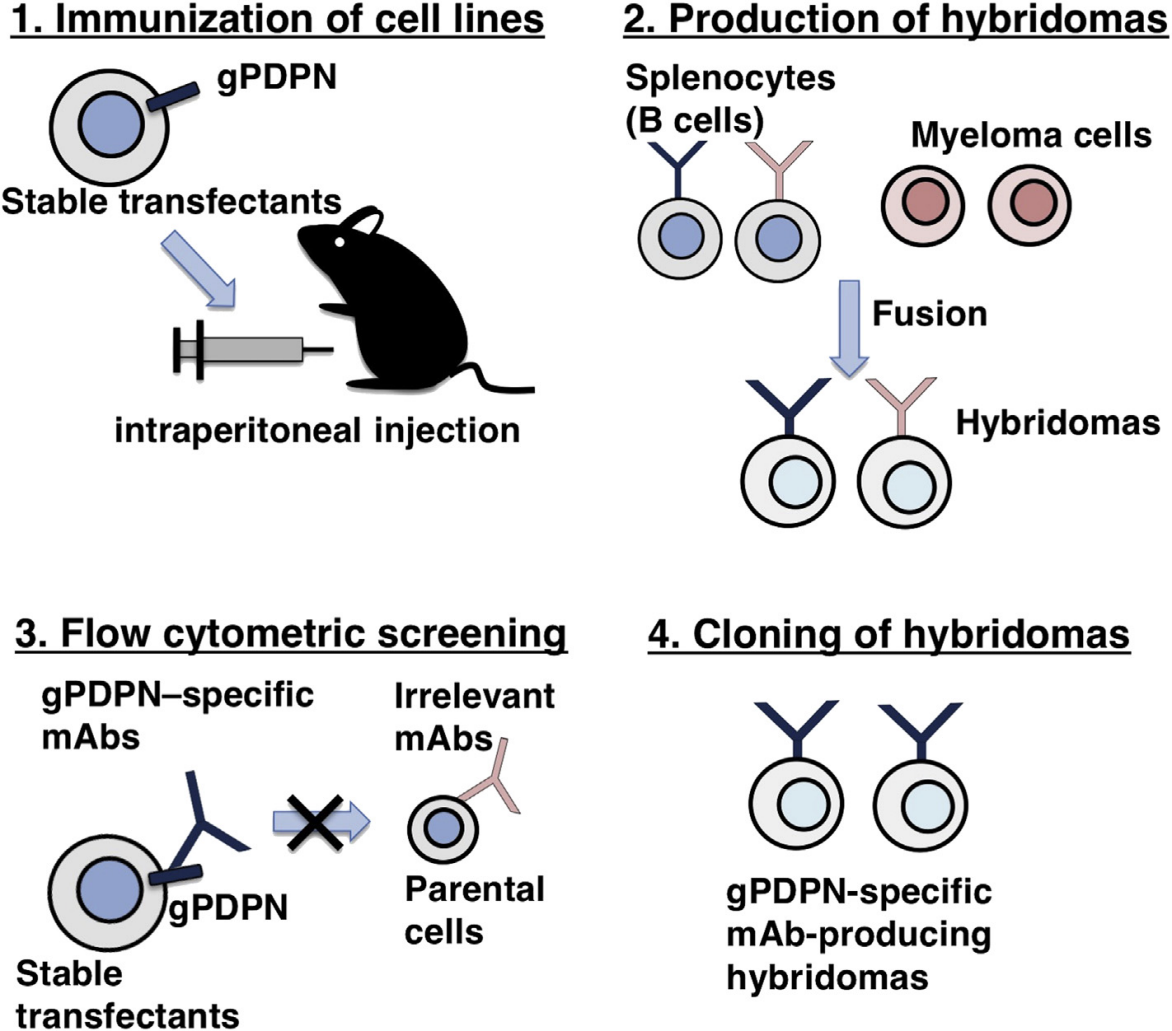
Schematic illustration of the Cell-Based Immunization and Screening (CBIS) method. Stable transfectants expressing the protein of interest are used as an immunogen with no purification procedure. The selection of hybridomas secreting specific mAbs is performed by flow cytometry using parental and transfectant cells.

**Fig. 2:**
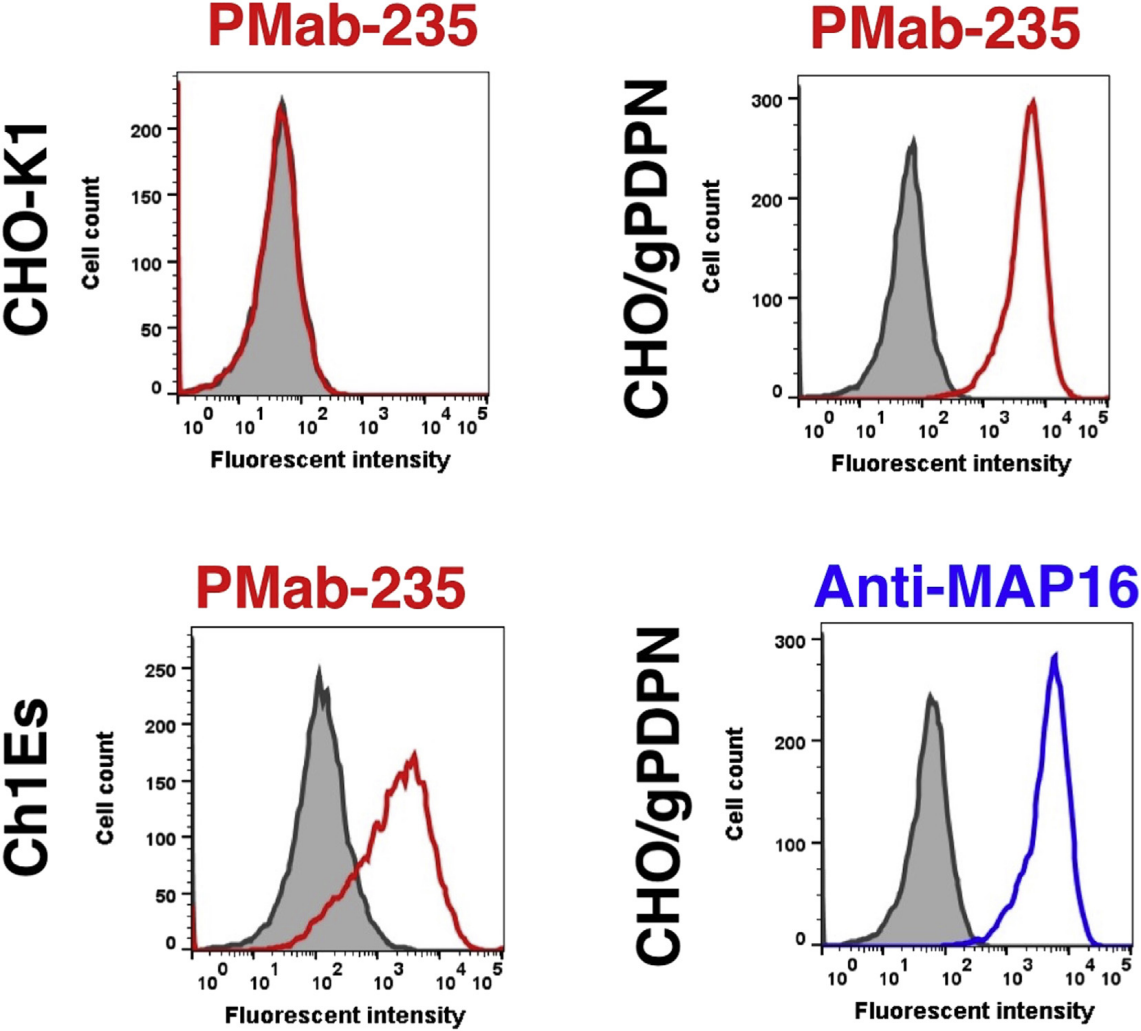
Detection of gPDPN by flow cytometry using PMab-235. CHO/gPDPN, CHO–K1, and Ch 1 Es cells were treated with PMab-235 (red line) or anti-MAP16 tag (PMab-1; blue line) at a concentration of 1 μg/mL or 0.1% BSA in PBS (gray) for 30 min, followed by incubation with secondary antibodies.

One mouse was immunized with CHO/gPDPN cells (Fig. 1). The developed hybridomas were seeded into 96-well plates and cultivated for 10 days. Wells positive for CHO/gPDPN and negative for CHO–K1 were selected by flow cytometry. The screening approach identified strong signals from CHO/gPDPN cells and weak or no signals from CHO–K1 cells in 29 of the 480 wells (6.0%). PMab-235 (IgG_1_, kappa) was finally selected via flow cytometry against Ch 1 Es, a fibroblastic cell line from normal fetal goat esophagus (Fig. 2). PMab-235 recognized CHO/gPDPN cells, but showed no reaction with CHO–K1 cells, as assessed by flow cytometry (Fig. 2). As a positive control, an anti-MAP16 tag (clone PMab-1) reacted with CHO/gPDPN.

**Fig. 3:**
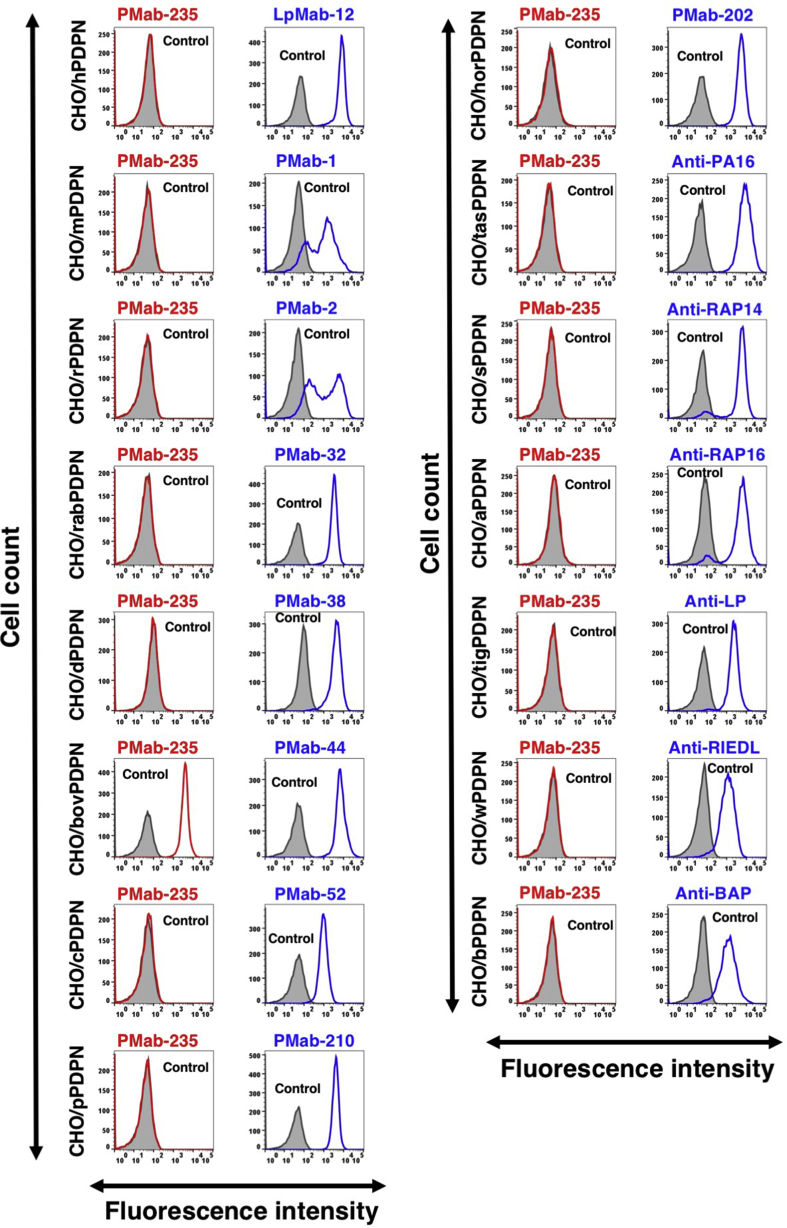
Cross-reaction of PMab-235 to PDPNs of the other species by flow cytometry. CHO–K1 cells transfected with PDPNs of the other species were treated with PMab-235 (red line) or each positive control (blue line) at a concentration of 1 μg/mL or 0.1% BSA in PBS (gray) for 30 min, followed by incubation with secondary antibodies.

We next investigated the crossreactivity of PMab-235 with PDPNs of the other species. PDPN expression levels in CHO cell lines were confirmed by positive controls, such as anti-PDPN mAbs or anti-peptide tag mAbs. We used clone LpMab-12 [36] as an anti-hPDPN mAb; clone PMab-1 [15] as an anti-mPDPN mAb; clone PMab-2 [16] as an anti-rPDPN mAb; clone PMab-32 [17] as an anti-rabPDPN mAb; clone PMab-38 [19] as an anti-dPDPN mAb; clone PMab-44 [18] as an anti-bovPDPN mAb; clone PMab-52 [20] as an anti-cPDPN mAb; clone PMab-210 [35] as an anti-pPDPN mAb; and clone PMab-202 [37] as an anti-horPDPN mAb. We also used clone NZ-1 as an anti-PA16 tag (GLEGGVAMPGAEDDVV) mAb; clone PMab-2 as an anti-RAP14 tag (DMVNPGLEDRIEDL) mAb; clone PMab-2 as an anti-RAP16 tag (GPGDDMVNPGLEDRIE) mAb; clone LpMab-17 as an anti-LP tag (NSVTGIRIEDLPTSES) mAb; clone LpMab-7 as an anti-RIEDL tag (RIEDL) mAb; and clone PMab-44 as an anti-BAP tag (EKTTLGVEDYTTTPAA) mAb. All positive control mAbs showed high reactivity for each PDPN-overexpressed CHO cell lines, such as human, mouse, rat, rabbit, dog, bovine, cat, pig, horse, Tasmanian devil, sheep, alpaca, tiger, whale, or bear PDPNs by flow cytometry (Fig. 3). PMab-235 crossreacted with only bovine PDPN, and did not react with human, mouse, rat, rabbit, dog, cat, pig, horse, Tasmanian devil, sheep, alpaca, tiger, whale, or bear PDPNs by flow cytometry (Fig. 3).

In addition, kinetic analysis conducted by flow cytometry was employed to assess the interaction of PMab-235 with CHO/gPDPN cells. *K*_D_ of PMab-235 for CHO/gPDPN cells was determined to be 1.5 × 10^−8^ M, indicating a moderate affinity of PMab-235 for CHO/gPDPN cells.

**Fig. 4:**
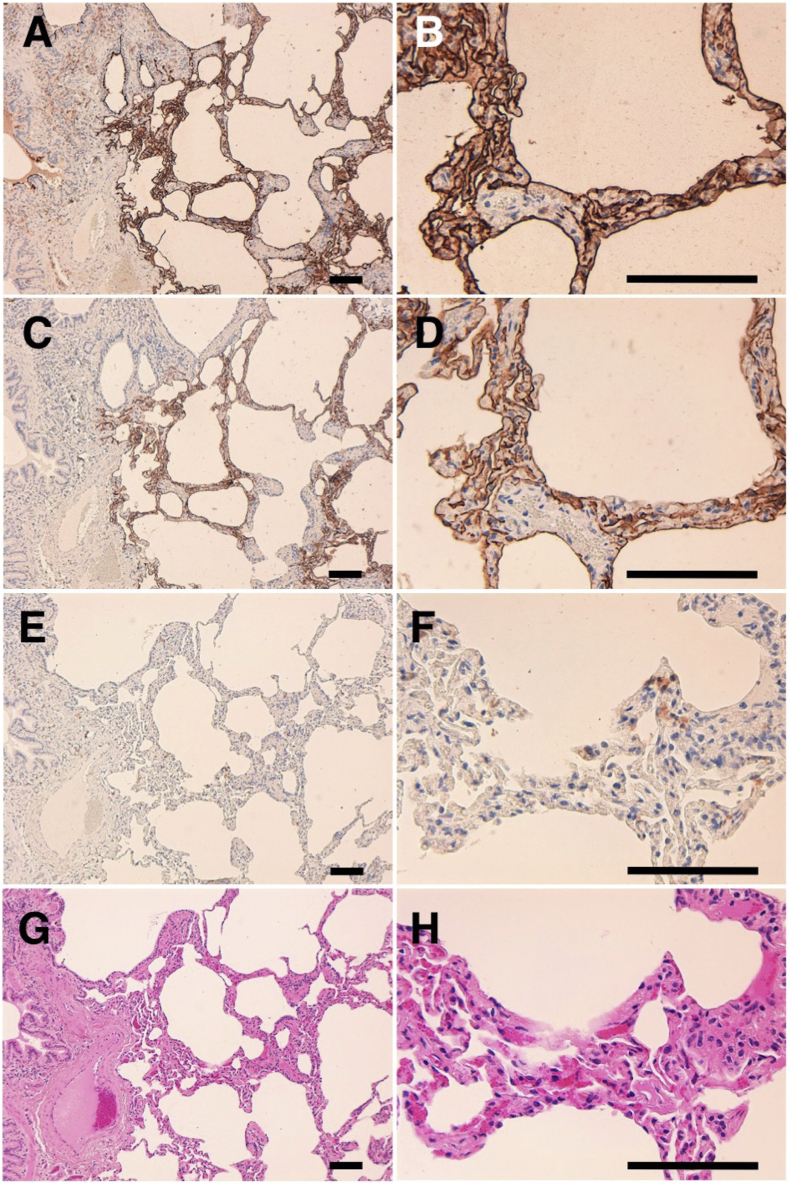
Immunohistochemical analyses for goat lung. Lung sections were incubated with 1 μg/mL of PMab-235 (A, B), 1 μg/mL of PMab-44 (C, D) or with blocking buffer (E, F), followed by that with the Envision + Kit. (G, H) Hematoxylin and eosin staining. Scale bar = 100 μm.

**Fig. 5:**
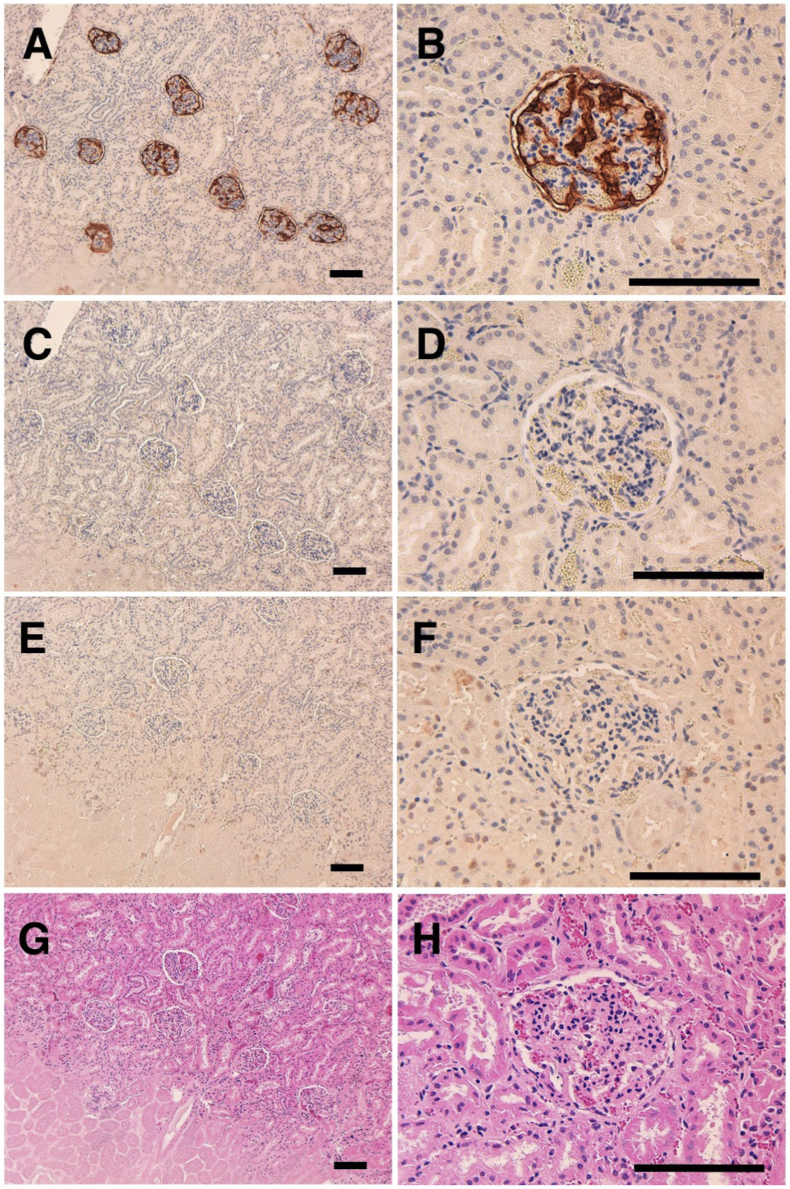
Immunohistochemical analyses for goat kidney. Kidney sections were incubated with 1 μg/mL of PMab-235 (A, B), 1 μg/mL of PMab-44 (C, D) or with blocking buffer (E, F), followed by that with the Envision + Kit. (G, H) Hematoxylin and eosin staining. Scale bar = 100 μm.

**Fig. 6:**
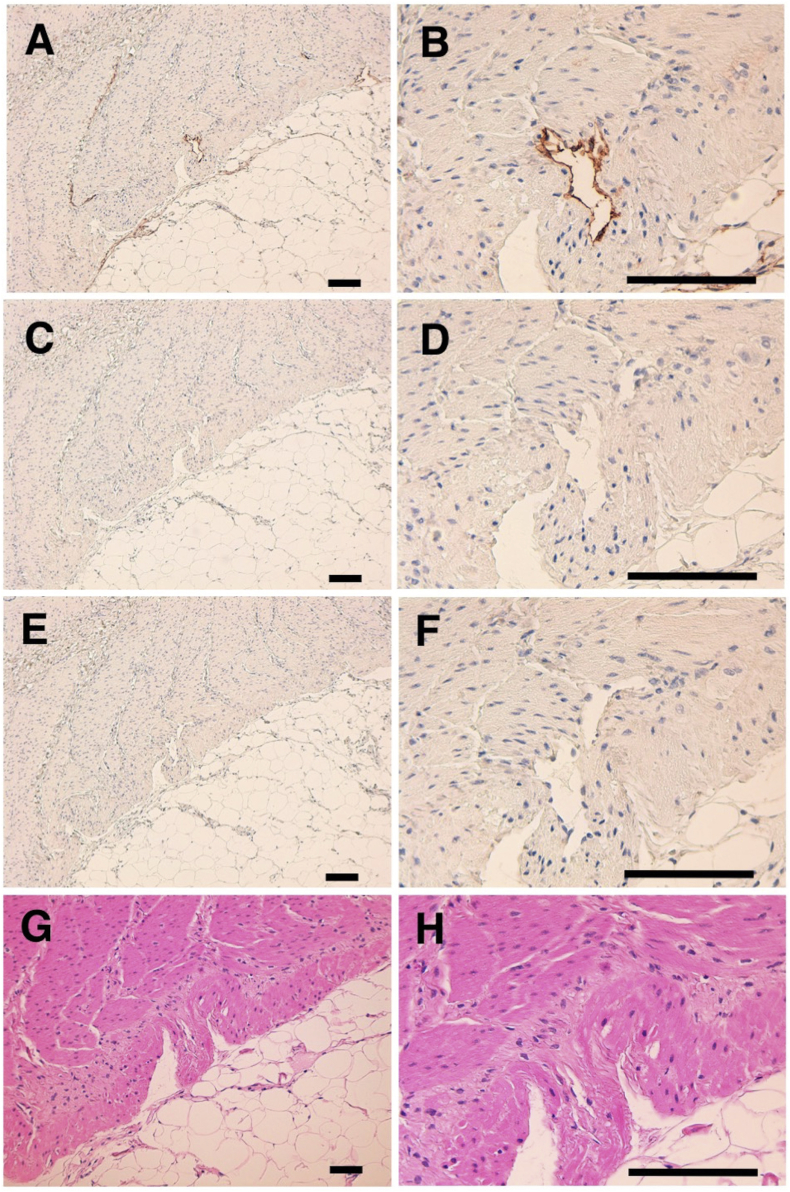
Immunohistochemical analyses for goat colon. Colon sections were incubated with 1 μg/mL of PMab-235 (A, B), 1 μg/mL of PMab-44 (C, D) or with blocking buffer (E, F), followed by that with the Envision + Kit. (G, H) Hematoxylin and eosin staining. Scale bar = 100 μm.

The immunohistochemical analyses revealed that PMab-235 strongly stained type I alveolar cells of lung (Fig. 4A and B), podocytes of kidney (Fig. 5A and B), and lymphatic endothelial cells of colon (Fig. 6A and B). We used PMab-44 as a positive control to stain gPDPN because PMab-44 was shown to crossreact with gPDPN in our previous study [38]. Although PMab-44 strongly stained type I alveolar cells of lung (Fig. 4C and D), it did not stain podocytes of kidney (Fig. 5C and D) and lymphatic endothelial cells of colon (Fig. 6C and D). No staining was observed without primary antibodies against type I alveolar cells of lung (Fig. 4E and F), podocytes of kidney (Fig. 5E and F), and lymphatic endothelial cells of colon (Fig. 6E and F).

## 4 Discussion

In 1979, Magari *et al.* reported morphological studies on liver lymphatics in human, pig, calf, dog, rabbit, and goat [39]. They studied form, distribution, and structure of liver lymphatics using both light and electron microscopes. Further, Ezeasor *et al.* reported the distribution and characteristics of lymph vessels in caprine (goat) hemal nodes after glutaraldehyde fixation and epoxy resin embedding [40]. Because anti-gPDPN mAbs, which are useful for immunohistochemical analysis to detect lymphatic endothelial cells, have not been reported, specific detection of lymphatic endothelial cells was difficult. Previously, we immunized mice with a synthesized peptide of gPDPN, such as _37_-KNEQTTLGVEDYMT-_49_, which is corresponding to platelet aggregation-stimulating (PLAG) domain. Unfortunately, we could not establish specific mAbs for immunohistochemical analysis against gPDPN (data not shown). Although an anti-bovine PDPN mAb PMab-44 crossreacted with gPDPN in immunohistochemistry for goat lung tissues, it did not react with lymphatic endothelial cells of goat tissues [38]. In the present study, we employed the CBIS method to develop sensitive and specific mAbs against gPDPN to facilitate the immunohistochemical analysis of paraffin-embedded tissue sections. Established PMab-235 reacted with endogenous gPDPN of a fibroblastic goat cell line as well as CHO/gPDPN cells (Fig. 2). The immunohistochemical analyses revealed that PMab-235 strongly stained type I alveolar cells of lung (Fig. 4), podocytes of kidney (Fig. 5), and lymphatic endothelial cells of colon (Fig. 6), indicating that PMab-235 is useful for the detection of gPDPN by immunohistochemistry. PMab-235 cross-reacted with bovine PDPN not only in flow cytometry (Fig. 3) but also in immunohistochemistry (data not shown). Unfortunately, PMab-235 did not react with gPDPN in western blot analysis (data not shown). In conclusion, we established PMab-235 against gPDPN, which is suitable for use in flow cytometry and immunohistochemical analyses using CBIS method. The epitope of PMab-235 needs further investigation to clarify the sensitivity and specificity of PMab-235 against gPDPN.

## Declarations

### Author contribution statement

Yoshikazu Furusawa: Performed the experiments; Wrote the paper.

Shinji Yamada, Takuro Nakamura: Performed the experiments.

Masato Sano, Shunsuke Itai, Junko Takei: Analyzed and interpreted the data.

Hiroyuki Harada, Masato Fukui: Contributed reagents, materials, analysis tools or data.

Mika K. Kaneko, Yukinari Kato: Conceived and designed the experiments; Wrote the paper.

### Funding statement

Yukinari Kato was supported in part by AMED (Grant numbers: JP18am0101078, JP18am0301010, and JP18ae0101028). Yukinari Kato was supported by JSPS KAKENHI (Grant Number 19K07705). Mika K.Kaneko was supported by JSPS KAKENHI (Grant Number 17K07299).

### Competing interest statement

Yukinari Kato received research funding from ZENOAQ RESOURCE CO., LTD.

### Additional information

No additional information is available for this paper.
